# Connecting Crop Productivity, Residue Fires, and Air Quality over Northern India

**DOI:** 10.1038/s41598-019-52799-x

**Published:** 2019-11-12

**Authors:** Hiren Jethva, Omar Torres, Robert D. Field, Alexei Lyapustin, Ritesh Gautam, Vinay Kayetha

**Affiliations:** 1 Universities Space Research Association, Columbia, MD 21044 USA; 2 NASA Goddard Space Flight Center, Greenbelt, MD 20771 USA; 3 Columbia University, NASA Goddard Institute for Space Studies, New York, NY 10025 USA; 4 Environmental Defense Fund, Washington, D. C., 20009 USA; 5 Science Systems and Applications, Inc. (SSAI), Lanham, MD 20706 USA

**Keywords:** Atmospheric chemistry, Environmental impact

## Abstract

Northwestern India is known as the “breadbasket” of the country producing two-thirds of food grains, with wheat and rice as the principal crops grown under the crop rotation system. Agricultural data from India indicates a 25% increase in the post-monsoon rice crop production in Punjab during 2002–2016. NASA’s A-train satellite sensors detect a consistent increase in the vegetation index (net 21%) and post-harvest agricultural fire activity (net ~60%) leading to nearly 43% increase in aerosol loading over the populous Indo-Gangetic Plain in northern India. The ground-level particulate matter (PM_2.5_) downwind over New Delhi shows a concurrent uptrend of net 60%. The effectiveness of a robust satellite-based relationship between vegetation index—a proxy for crop amounts, and post-harvest fires—a precursor of extreme air pollution events, has been further demonstrated in predicting the seasonal agricultural burning. An efficient crop residue management system is critically needed towards eliminating open field burning to mitigate episodic hazardous air quality over northern India.

## Introduction

Crop residue burning over northwestern India is a serious concern leading to poor air quality and affecting the health of millions living in one of the most densely populated regions of the world. The issue has received a great deal of attention after a consistent ranking of several major cities in the Indo-Gangetic Plain (IGP), including New Delhi, in the WHO reports having the poorest air quality related to particulate matter. A 15-year long record (2002–2016) of NASA’s A-train satellite measurements have revealed a positive trend in the total fire activity and resulting aerosol loads over IGP. This study investigates the probable cause of rising agricultural fires and deteriorating air quality over the region. Increasing agricultural fire activities imply greater availability of crop residue to burn, and the generation of waste is proportional to the crop production amounts. Our work verifies this hypothesis by quantifying the link connecting the crop production followed by residue fires and air quality measures using a suite of satellite and ground observations.

## Major Findings

Our study finds that rice production in the northwestern state of Punjab has increased by 25%, and so has the vegetation index (NDVI) with a net increase of 21% derived from the MODIS sensor onboard Aqua satellite during 2002–2016. The amount of agricultural waste generated post-harvest is estimated to be 1.5 to 2.25 times the actual quantities of crop, as reported by the earlier studies. Due to the lack of affordable and effective removal mechanism, farmers resort to burning crop residue in open fields to clear and prepare the land for winter wheat crop. The Thermal Anomaly product of MODIS reveals a concurrent increase of 60% in agricultural fire activity, and further exhibits a clear relationship with the vegetation index during 2002–2016. The reconstructed time-series of PM_2.5_ derived based on a regression between the ground-level particulate matter concentration visibility estimates in New Delhi has shown a simultaneous uptrend of 6 µg/m^3^ per year, leading to 60% increase during post-monsoon season. The crop burning season of 2016 has been the most anomalous with the largest estimates of crop production followed by the maximum numbers of crop fires in northwestern India and record-breaking levels of PM_2.5_ readings in New Delhi. The impact of agricultural fires is not just restricted to the proximate areas, but encompasses the entire IGP as revealed by the consistent positive trends (~43%) in the satellite measurements of aerosol loading. A robust relationship between vegetation index—a proxy for the crop productivity, and post-harvest accumulated fire activity—a precursor of poor air quality, allows the prediction of the intensity of crop fire season and the resulting degradation of air quality in advance, the effectiveness of which is successfully demonstrated for the harvesting seasons of years 2017 and 2018.

## Crop Residue Burning and Air Quality Link

Particulate matter (PM) and trace gases emitted from the open field agricultural burning have a high potential to alter the radiation balance of Earth, trigger changes in atmospheric chemistry, and can severely affect local and regional air quality. The 2014 report of the World Health Organization (WHO)^1^ states that 7 million deaths-one in eight of total global deaths were linked to air pollution in 2012, and confirmed that air pollution is the world’s largest single environmental health risk. PM_2.5_-a major component of the overall air quality measure^1^, is known to exert detrimental effects on human health. About 7,350–16,200 premature deaths and 6.0 million asthma attacks per year have been attributed to the observed PM_2.5_ levels (averaged 123 ± 87 μg/m^3^) in New Delhi^2^. Furthermore, the exposure to PM_2.5_ is estimated to result in about 570,000 premature deaths in India with IGP accounting for a large part of the estimated mortalities^3^.

In recent years, New Delhi and other cities in IGP—a densely populated low-lying area in northern India bounded by topography in north-south, have been consistently ranked among the most polluted cities in the world^4,5^. The severe haze event of post-monsoon 2016 over northern India observed a record high-level mass concentration of PM_2.5_ (two-week averaged 440 ± 265 µg/m^3^) at the U.S. Embassy in New Delhi in the last (first) week of October (November). The hazardous level of PM_2.5_ concentration was 20 to 37 times above the safe guideline value of PM_2.5_ (24-hour averaged 25 µg/m^3^) set by WHO, leading to a public health emergency; the factor was 8–15 when compared against the standards adopted by the Central Pollution Control Board of India (24-hour averaged 60 µg/m^3^). During the same time, the crop residue fires in the northwestern Indian states of Punjab and Haryana detected from MODIS onboard Aqua satellite were also unprecedently high^6^. The National Capital Region (NCR) of Delhi is known to be severely affected by the crop waste burning in the neighboring Indian states^6–10^. Furthermore, the northwesterly wind flow distributes carbonaceous smoke particles generated from the crop residue burning region of Punjab and Haryana states to over downstream areas of IGP leading to poor air quality over the entire region^6,7^.

Under the Wheat-Rice-Crop-Pattern (WRCP), the northwestern region undergoes two major growing seasons: one from May to September followed by rice harvesting during October-November, and another from November to April followed by wheat harvesting in April-May. Since the mid-1980s, the practice of manual harvesting has been replaced by the advent of automatic combines^11^ that leaves a significant portion of the crop stem root-bound. Due to the lack of affordable crop residue removal mechanisms (i.e., easy-to-access tools and machinery that farmers can bear financially for residue removal) and given a shorter time window for preparing the land for the next crop, the residue is subjected to burning in open fields^12^. Our analysis of the MODIS fire detection product shows that, on an average (2002–2016), post-monsoon crop fires in these two states account for about 84% (19%) of the total fire hotspots detected over the entire Indian subcontinent (8°N-35°N, 68°E-95°E) seasonally (annually).

The PM_2.5_ readings in New Delhi have been shown to correlate with NASA’s A-train satellite measurements of post-monsoon fire activities and aerosol loads in Punjab-Haryana^6^. The satellite record has also revealed positive trends in fire activity and aerosols over IGP. The present study further investigates the possible cause of increasing crop fires and aerosol loading over the region using satellite datasets, ground-level PM_2.5_ measurements, and as a longer-term PM_2.5_ proxy, horizontal visibility reported at airports. Increasing agricultural fires imply the availability of more crop residue to burn, and the generation of waste is proportional to the crop production amounts. We evaluate this hypothesis by quantifying the link between them and propose a practical approach to predict the severity of the burning season in advance.

## Results

### Temporal evolution of crop production, vegetation index, and fire activity

**Figure 1:**
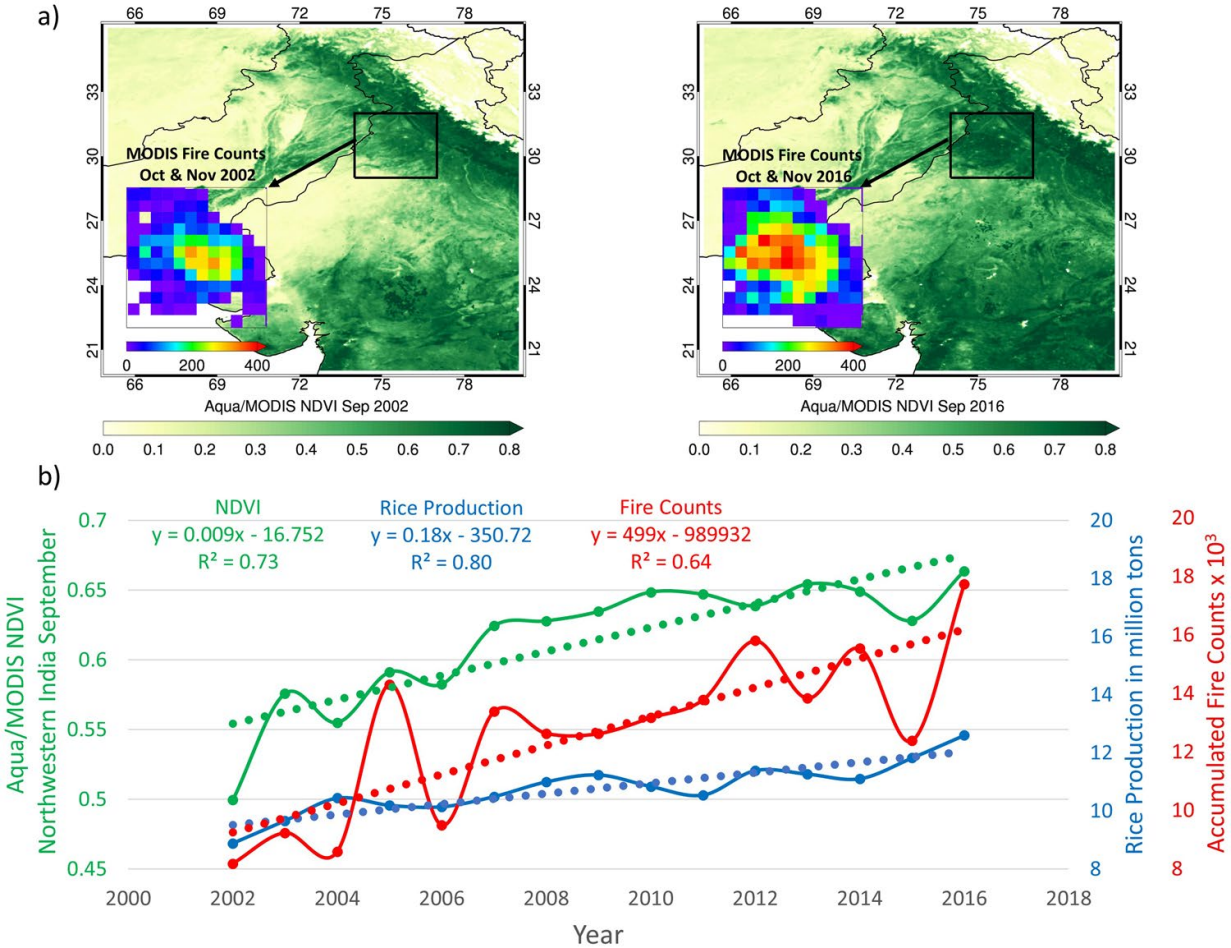
(**a**) Satellite detection of NDVI and corresponding fire activity for 2002 and 2016, and (**b**) interannual changes in rice production, NDVI, and seasonally accumulated fire counts over northwestern India. NDVI data from Aqua/MODIS MYD13C2 product represents the surface condition for September before the onset of crop burning, whereas the accumulated fire counts are detected during the crop burning months of October and November. The dotted lines in the bottom chart represent linear trends calculated from the respective monthly datasets. The region of northwestern India is shown as a bounded grid box with longitude: 74°E–77°E and latitude: 29°N–32°N. We used Interactive Data Language (IDL, https://www.harrisgeospatial.com/docs/using_idl_home.html) software version 8.7.2 to prepare both maps showing NDVI and fire activities.

Satellite maps of the Normalized Difference Vegetation Index or NDVI pre-harvest and accumulated agricultural fires for the harvesting season of 2002 and 2016 displayed in Fig. 1(a) highlight a marked difference in the “greenness” of crop fields relating to the crop productivity and subsequent intensity of residue fires over northwestern India. The multi-year evolution of rice production and concurrent September NDVI values derived from Aqua/MODIS over northwestern India shown in Fig. 1(b) reveal an overall increase during 2002–2016, yielding positive linear trends of 0.18 million tons and 0.009 per year leading to an overall 26% and 21% increase, respectively. A steady increase in rice crop production has been a result of growth in both cultivated area and yield (Supplementary Fig. 1). The coherent temporal changes in both quantities are consistent with the findings of previous studies demonstrating the effectiveness of NDVI as a proxy of the crop production amounts^13–16^.

Naturally, the increases in crop yields produce more residue, which farmers in northwestern India traditionally burn in open fields to clear and prepare the land for the next crop. The residue to crop production ratio (RCR), as estimated from several studies, varies considerably depending on crop type, harvesting practice, and environmental factors^17^, Table 2.4. For instance, the harvest of 9.9 million tons of rice crops over Punjab results in approximately 18.75 million tons of crop residue–an RCR of 1.89^18^. A rice residue amount in the range of 6.2–11.8 million tons/hectare is reported in^12^, which by assuming a crop yield of 4 million/hectare results in an average RCR of 2.25. Every 4 tons of rice or wheat grain is stated to produce about 6 tons of residue straw equating RCR to be 1.5^19^. Regardless of the spread of RCR estimates, these studies suggest that residue amounts are considerably larger than the actual crop production.

The multi-year time-series of seasonally accumulated fire counts detected by MODIS over Punjab-Haryana show an upward trend of ~500 fire detections per year, leading to a net 60% increase during the Aqua satellite record. The post-harvest season of 2016 encountered an unprecedented fire activity (17,804) concurrent with the highest rice production (12.6 million tons) and highest NDVI (0.66) reported for the pre-burning month of September. The average fire activity detected during the second half of the Aqua record (2009–2016) turns out to be 30% higher against that of the first half (2002–2009) —an uptrend consistent with a distinct increase in burned area over the same region from 1998 to 2015^20,21^.

**Figure 2:**
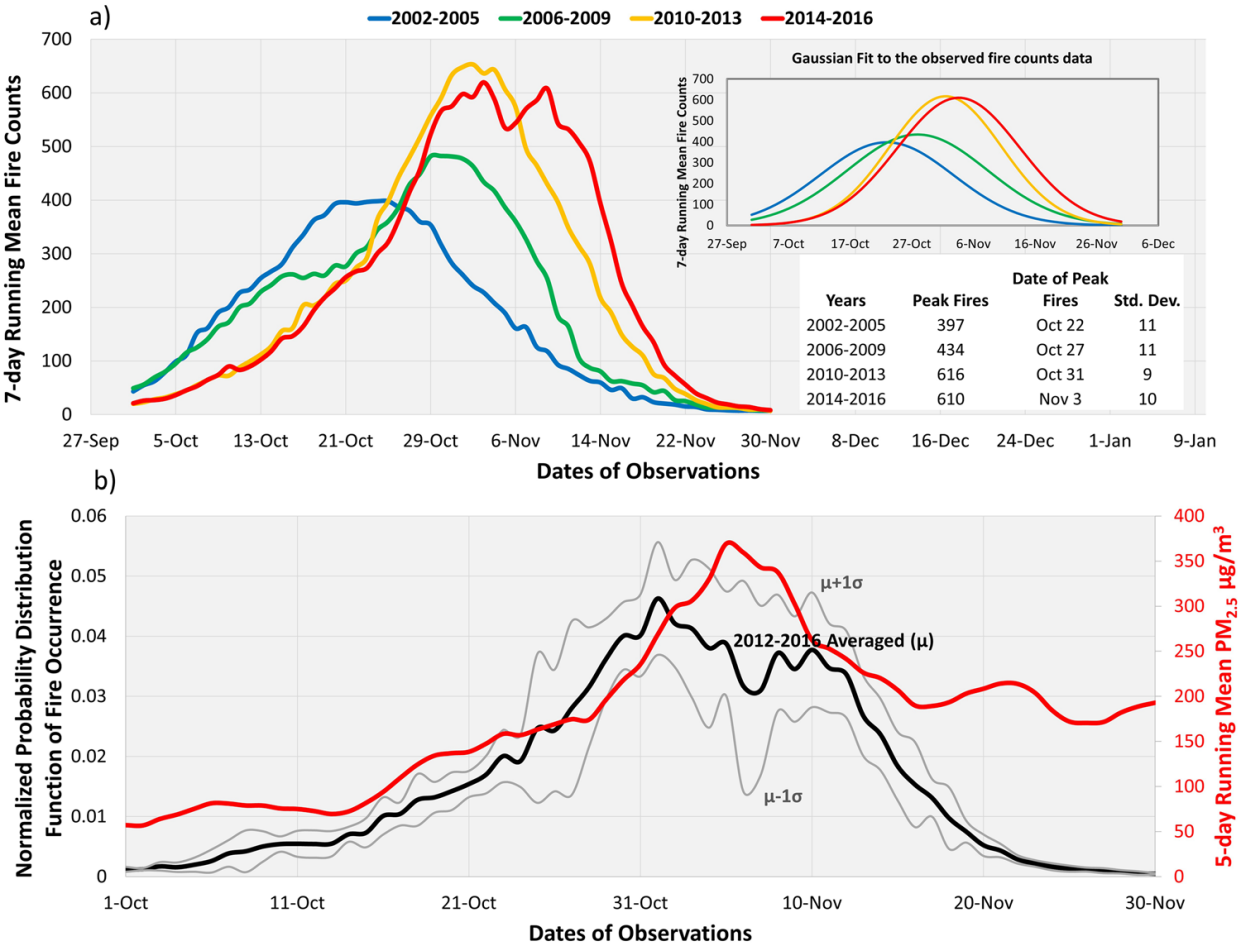
Sub-seasonal evolution of satellite-detected fire counts (**a**), normalized pattern of fire occurrence (**b**, left), and daily-averaged PM_2.5_ in New Delhi (**b**, right) during 2012–2016. The Gaussian fits to the observed fire count data are shown in the inset with parameters of the distribution tabulated in the lower-right of top chart. The Probability distribution function (PDF) of fire counts normalized to the total number of fires during the respective years (20120–2016) is shown in the inset; averaged (μ) PDF with the corresponding 1-standard deviation (σ) derived using the recent five years of data are shown in black ink.

In 2009, the state government of Punjab had enacted a law called as the “Punjab Preservation of Subsoil Water Act, 2009”^22^, requiring that farmers delay the nursery sowing and transplantation of paddy after May 15 and June 15, respectively. The intentional delay in transplanting of rice until mid-June is aimed at saving groundwater resources by aligning the rice-growing season with the Indian summer-monsoon cycle. The enforcement brought by this Act has led to the delay in crop harvesting followed by the residue burning. The intra-seasonal evolution of fire activity shown in Fig. 2(a) reveals that the density of the seasonal fire counts can be closely approximated as a Gaussian distribution with a peak in the fire activities shifting from 3^rd^ week of October to 1^st^ week of November. Though the Act was brought into force since 2009, a shift in the timing of peak fire activities took place earlier during 2006–2009 after which the majority of fires further delayed by about a week during 2010–2016, as also noted in^23^.

### Crop Productivity-NDVI-Fire density relationship

**Figure 3:**
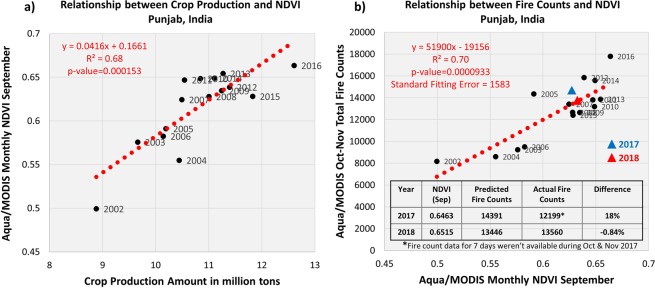
Relationship between the crop amounts and pre-burning season NDVI (left), and pre-burning season NDVI and the total number of fire counts during crop burning months (right). Both datasets are derived from the Aqua/MODIS sensor over northwestern India. NDVI serves as a proxy of crop production, and total fire counts are indicative of crop residue amounts burned in the open fields. The coefficients of the linear regression fit (red dotted line), correlation, and standard fitting error are depicted at the top-left. A table included on the right lists NDVI, predicted and actual fire counts, and their differences in percent for the burning seasons of years 2017 and 2018.

Fig. 3(a) shows that NDVI is related to crop production, which is expected to produce crop waste in proportional amounts. Although both quantities are fairly correlated with each other in long-term (R^2^ = 0.68), their interannual variabilities appear to be inconsistent during specific years. It is expected that the crop productivity estimations are subjected to greater uncertainties than the satellite measurements of NDVI. This is because it isn’t a directly measured quantity and involves several steps and datasets for the inference, including satellite detection of vegetation indices, estimation of area coverage, ground-truthing, the calibration that relates these measurements to crop production, etc. These factors have likely contributed towards incoherent interannual variations in both datasets.

About 80% of the agricultural leftover from rice harvesting is burned in open fields post-harvest in Punjab^16^. Therefore, a close association is expected between the pre-burning NDVI and post-harvest residue fires. Such a relationship, as shown in Fig. 3(b), would be useful to estimate the intensity of crop fires post-harvest given prior knowledge of NDVI before the onset of burning. Several factors could have contributed to the observed spread in the relationship, including inherent uncertainties associated with the detection of fire counts and derivation of NDVI, hindrance of cloud cover affecting sampling, unaccounted fire activities occurring before or after Aqua overpass time, a saturation of NDVI in dense vegetation canopies introducing non-linearity in the relationship, and variabilities in RCR and percentage of total crop residue burned. Despite these limitations, a robust NDVI-fire counts relationship is useful to predict an estimate of the seasonally accumulated crop fires. The anticipated total fire activity can be further distributed within the two-month-long fire season based on the averaged sub-seasonal pattern of burning observed during the recent five years (2012–2016) as shown in Fig. 2(b). The concurrent variations in PM_2.5_ in New Delhi can also be estimated by correlating its temporal pattern to the simultaneous behavior of predicted crop fires. Such prediction capability can serve as a guideline for the poor air quality related planning and preparedness.

To test the efficacy of the derived fire activity-NDVI relationship, the total number of probable fire detections was estimated for the crop burning season of 2017 and 2018 based on the respective September month NDVI values. It is noted here that data from these two latest years were not included in the 2002–2016 regression analysis. The predicted fire counts are found to agree well with the MODIS observations with a difference of 18% and −0.84% for the years 2017 and 2018, respectively. The larger error in prediction for the year 2017 is partly attributed to the non-availability of satellite data for a total of 7 days resulting in the undersampling of actual fire hotspots. A robust agreement with only −0.84% difference between the predicted and observed fire activity for 2018, when continuous observations from MODIS are available, suggests the effectiveness of the proposed approach involving crop production and NDVI in predicting seasonally accumulated agricultural fire activity.

With the implementation of the effective crop residue management system, one may expect a decrease in the frequency of residue burning. Under this scenario, the NDVI-fire density relationship would produce significant departure of the predicted fire counts from actual observations. Conversely, significant errors beyond the inherent uncertainties associated with the predictability of fire season can further help to evaluate the effectiveness of residue management intended towards reducing fire activities and resulting air pollution.

### Implications for air quality

#### Reconstruction of PM_2.5_ time series

**Figure 4:**
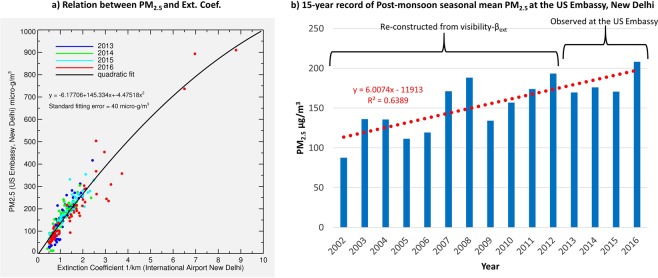
Relationship between daily-averaged PM_2.5_ and extinction coefficient (left) and interannual time-series of seasonal mean PM_2.5_ calculated from the visibility record in New Delhi. The daily-averaged PM_2.5_ was calculated using observations acquired between hours 5:00–24:00 local time at the US Embassy in New Delhi and extinction coefficient calculated from the visibility recorded at the Indira Gandhi International Airport, New Delhi for the period 2013–2016. The solid black line represents a quadratic fit to the daily data matchups with coefficients and standard fitting error depicted at top-left.

A contemporary overlap of PM_2.5_ measurements and visibility reports at two nearby sites (~9 km apart) in New Delhi allows a direct comparison for establishing a relationship between them, such as shown in Fig. 4(a). PM_2.5_ is related to β_ext_ through a quadratic fit with a standard error of 40 µg/m^3^, which reflects the imperfectness in the assumption that the two quantities measured at two different stations apart by ~9 km are correlated. While most matchups are concentrated in the β_ext_ range 0.5–2.5 km^−1^ corresponding to the PM_2.5_ range 0–300 µg/m^3^, a few observations of the extremely poor air quality event of the year 2016 extends the relationship to a much broader scale. Although the visibility observations are subjective and carry uncertainties that depend on the skill of the human observer and local settings, its well-defined relationship to PM_2.5_ via β_ext_ offers a practical way of estimating long-term time-series of PM_2.5_. The daily-averaged PM_2.5_ at New Delhi reconstructed from the visibility-β_ext_ observations combined with actual measurements of PM_2.5_ displayed in Fig. 4(b) show an increasing trend of ~6 µg/m^3^ (4%) per year (total 60%) over the period 2002–2016. The average value of PM_2.5_ for the second half of the time-series (2009–2016) is noted 28% higher than that from the first half (2002–2009)—an increase consistent with the 30% uptrend in fire counts over the crop burning region upwind in the northwest. Despite the inherent uncertainties involved in both PM_2.5_ and visibility datasets and methodology correlating them, a long-term trend in the reconstructed PM_2.5_ consistent with the crop fires upwind demonstrates the effectiveness of the present approach.

#### Long-term trends in air quality

**Figure 5:**
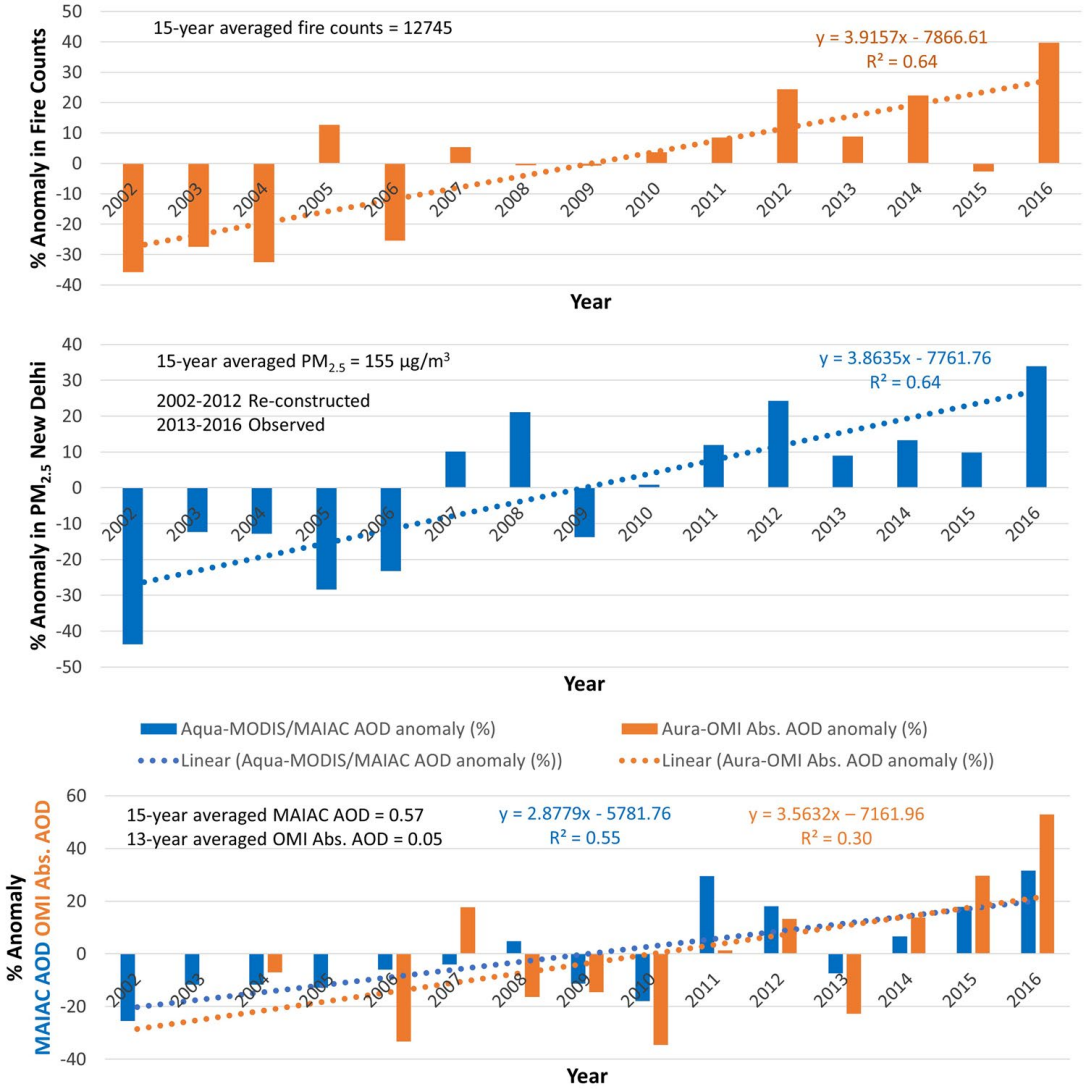
Interannual changes of the anomaly (%) in satellite detection of fire counts, reconstructed PM_2.5_, aerosol loadings for the post-monsoon season. Fire count data from Aqua/MODIS were considered over the crop burning areas, whereas measures of aerosol loading (extinction and absorption AOD) encompass broader Indo-Gangetic Plain. PM_2.5_ data were consolidated by combining the reconstructed record (2002–2012) and actual measurements made at the US Embassy in New Delhi. The percent anomalies were calculated against the long-term averages, as depicted in the chart, in respective quantities.

Observations spanning the past decade and half of the fire activity and aerosol loading from A-train satellite data along with the reconstructed record of PM_2.5_ offer a unique opportunity to assess the trends in crop burning and particulate matter. Fig. 5 presents the % anomaly of the fire counts for the Punjab-Haryana region, PM_2.5_ in New Delhi, MODIS MAIAC AOD, and OMI absorption AOD for the broader area of IGP (see Methods). The anomalies were calculated by subtracting the long-term averaged value from the seasonal mean values of the respective parameters. A common striking feature in all three time-series is the persistence of negative (positive) anomalies during the first (second) half of the A-train record, suggesting that the fires and aerosol loads, including ground-level PM_2.5_, have steadily increased where the year 2008–2009 appears as a crossover point. The crop burning season of 2016 had the highest number of fire count detection, with record-high PM_2.5_, extinction AOD, and absorption AOD reaching a maximum level at about 40%, 35%, 30%, and 50% from the long-term mean respectively. The overall absolute trend in these parameters was noted as 500/year, 6 µg-m^−3^/year, 0.019/year, and 0.001/year, respectively.

**Figure 6:**
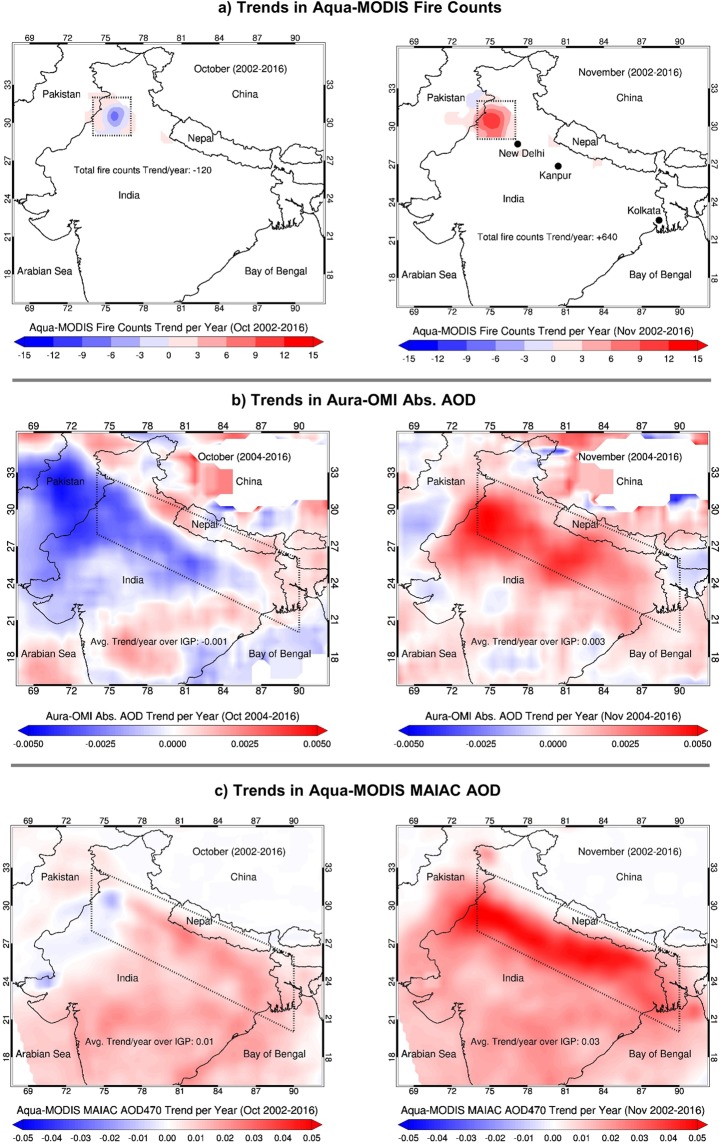
Spatial patterns of long-term trends (2002–2016) in fire counts and aerosol loading over most of the Indian subcontinent. Trends per year for October are shown in left-side panels; for November in right-side panels. Fire count and MAIAC-retrieved AOD datasets belong to Aqua/MODIS, and absorption AOD to Aura/OMI. Trends were calculated using the monthly gridded datasets for their respective periods. The crop burning region of northwestern India is shown as a box.

The spatial distributions of multi-year trends in these parameters are shown in Fig. 6. Fire count data from MODIS reveals a decreasing trend of −120 fire spots/year (−1.62%/year) in October, whereas fire activities in November have shown a drastic increase by +640 fire spots/year (+10.63%/year). Concurrently, the trends in absorption and extinction AOD for October over the crop burning region show a decrease by −3.73%/year and −0.12%/year, respectively, consistent with the declining trends in residue fires. Overall, the trend in extinction AOD is found to be marginally positive (+1.74% AOD/year) over the broader IGP, which is mainly driven by an increase in aerosol loading outside the crop burning region and over the eastern parts of IGP. In contrast, the aerosol trends in November are significantly positive over the entire stretch of IGP resulting in area-averaged trends of 0.003 (2.86%) and 0.03 (3.71%) per year in absorption and extinction AOD, respectively. Similarly, large positive trends in satellite extinction AOD for November are noticed over major urban locations in the IGP, i.e., New Delhi (5.1%/year), Kanpur (4.0%/year), and Kolkata (3.79%/year) that are in the path of smoke transport downwind of the burning region (Supplementary Fig. 2b). The ground-based AERONET site in the center of IGP at Kanpur further reveals a consistent 34% increase in the AOD (~0.03/year) during 2002–2016 (see Supplementary Fig. 3).

With respect to the seasonality of aerosol loading over IGP, the MAIAC AOD dataset showed the largest uptrend of 45% during post-monsoon agricultural burning months, followed by 40%, 11%, and 15% increase during winter, spring, and monsoon seasons respectively (Supplementary Fig. 4). A distinct rise in aerosol amounts post-monsoon coincident with the increasing crop residue burning in the northwest suggests that the latter is one of the main emission sources responsible for deteriorating air quality over northern India.

### Final remarks

Our study has established, using the long-term measurements from satellites and ground sensors, a strong connection between the increasing crop production and thus residue amounts, fire activity, and resulting particulate matter pollution over the entire breadth of IGP. A robust empirical approach correlating the satellite-based observations of vegetation index and fire activity offers a practical way to predict the seasonal agricultural burning and its evolution that can deliver actionable data towards mitigating transient yet extreme air pollution sources. The wealth of satellite observations has made possible the long-term monitoring of proxy for crop amounts, fires, and air pollution and associated trends derived in this study. While the increasing agricultural productivity is favorable for the nation’s food security, the practice of crop waste burning significantly contributes to extreme levels of air pollution in the region causing hazardous conditions and affecting the health of millions. Rising levels of crop fires and deteriorating air quality over IGP is a serious concern calling for the implementation of an effective crop residue management system, the lack of which may likely lead to the continuation of crop waste burning and the resulting degradation of air quality over northern India.

## Methods

### Crop production data

The rice crop production data were accessed from the Crop Production Statistics Information System (http://aps.dac.gov.in/APY/Index.htm) designed and developed by the Agriculture Informatics Division, National Informatics Centre, Ministry of Communication & IT, Government of India. The crop production data are compiled by the Directorate of Economics and Statistics, Ministry of Agriculture and Farmers Welfare, Govt. of India (http://eands.dacnet.nic.in/). The dataset contains the crop statistics including cultivated area (hectares), yield (kg/hectare), and production amounts (million tons) for a variety of crops grown in every district and state of India. These estimates are derived using remote sensing approaches employing observations mainly from NOAA-AVHRR (for drought assessment), Terra/Aqua-MODIS (for Rabi season crop alert), Resourcesat 2- AWiFS & LISS III (for Wheat), and RISAT-1 SAR (for Rice) in conjunction with ancillary data and ground-truth^24^. We extracted the rice crop statistics for the state of Punjab for the *Kharif* season (June to September) after which the crop is harvested in October and November. The rice crop production data shown in Figs 1(b) and 3(a) cover the period 2002–2016.

### Ground-level PM_2.5_ measurements

The US Embassy & Consulates operate PM_2.5_ measuring instrument (i.e., MetOne BAM 1020), under the Air Quality Monitoring program in the five metro cities of India, including New Delhi. The PM_2.5_ measurements carried out at foreign locations are operated by the U.S. Department of State in collaboration with the U.S. Environmental Protection Agency (EPA). The MetOne BAM 1020 unit measures the concentration of PM_2.5_ of the ambient air sample on an hourly basis following the Beta attenuation method. The hourly PM_2.5_ data for 2013 to 2016 taken at the U.S. Embassy site in New Delhi were accessed from a publicly accessible URL https://in.usembassy.gov/embassy-consulates/new-delhi/air-quality-data/. The daily-averaged PM_2.5_ displayed in Fig. 4 was calculated using hourly data between 5:00–24:00 local time measured at the US Embassy in New Delhi for the period 2013–2016.

### Visibility data from NOAA’s climate data center

The horizontal visibility record is used as a proxy for air quality prior to the year 2013 when PM_2.5_ measurements began in New Delhi. Among several factors, particulate matter in the atmosphere between the observer and the object is a significant factor that can affect visibility. Poor air quality contributes to reduced visibility since the air laden with particles attenuates the visible light through scattering and absorption. Visibility records have been used to constrain biomass burning contributions to aerosol loading in northern Australia^25^, Indonesia^26–28^, and South America^29^. Visibility records have also been used to understand drivers of interannual variability in dust^30^ and to identify trends in global atmospheric haze more generally^31^. Following these studies, visibility reports were used to estimate the extinction coefficient (βext) using the empirical Koschmeider relationship βext = 1.9/visibility, where visibility is reported in kilometers^32^.

The extinction coefficient βext was calculated using visibility reports for Indira Gandhi International Airport (station ID: 421810) in New Delhi obtained from the Integrated Surface Database (ISD) at NOAA’s National Center for Environmental Information. Next, the PM_2.5_ observations from the US Embassy were related to βext for a contemporary overlap period of 2013–2016. Visibility reports flagged as having suspect quality in the ISD records and/or where relative humidity was >90% were excluded to lessen the influence of fog^32^. The data shown in Fig. 4 are daily-averaged PM_2.5_ (y-axis) versus extinction coefficient β_ext_ (x-axis). The latter was calculated using visibility observations carried out at the primary synoptic reporting times of 5:30, 11:30, 17:30, and 23:30 local time to maintain a consistent reporting frequency, whereas the former was averaged using measurements between 5–24 hours local time to match with the times of visibility observations.

### Aerosol robotic network (AERONET)

The long-term direct measurements of spectral AODs from the ground-based AERONET—an international federated network of sunphotometers^33^, at Kanpur (longitude: 80.40°E, latitude: 26.45°N), India were considered as a ground-truth to validate the MODIS-MAIAC retrieval of AOD (Supplementary Fig. 3a), and also used to calculate the multiyear trend in AOD (b).

### NASA’s A-train satellite retrievals

#### MODIS normalized difference vegetation product

MODIS vegetation indices produced on 16-day intervals and at multiple spatial resolutions are derived from atmospherically-corrected surface reflectance in the red (670 nm) and near-infrared (860 nm) wavelength bands. We use Aqua/MODIS Collection 006 monthly, Level 3 global NDVI dataset (MYD13C2) for the period 2002–2016^34^. The MYD13C2 dataset consists of cloud-free spatial composites of the gridded 16-day, 1-km product (MYD13A2) and provided on a monthly scale as a Level-3 product projected on 0.05° grids. The dataset was accessed from LP DAAC online data holdings at https://e4ftl01.cr.usgs.gov/MOLA/. The remotely sensed NDVI, derived based on the spectral contrast in the red and near-infrared (NIR) wavelength bands, is commonly used to estimate the “greenness” of land cover^13^. Higher values of NDVI are related to the increased greenness of land cover, such as when crop fields are at the maturity stage. Several India-specific studies^14–16^ have shown that NDVI offers a practical approach to estimate crop production as it directly relates to the potential photosynthesis activities and various stages of crop growth. The increased greenness of the cropland due to the maturity and/or greater cultivated area produces a marked contrast between the reflectance in the red and NIR bands resulting in higher magnitudes of NDVI. The monthly area-averaged NDVI values shown in Figs 1 and 3 are scaled by a factor that accounts for interannual variations in the spatial extent of NDVI measurements over the crop area. The factor was calculated by normalizing the total number of MODIS 1-km pixels used for the NDVI calculations for each year with respect to the maximum number of pixels detected during a particular year, which turns out to be the year 2016.

#### MODIS thermal anomaly/fire product

The Thermal Anomaly/Fire product of MODIS provides the geolocation of active fire spots over land at 1 × 1 km^2^ spatial resolution globally. The fire detection is performed using a contextual algorithm that exploits the strong emission of mid-infrared radiation from fires. A detailed description of the MODIS fire detection algorithm is given in^35,36^ and in the Algorithm Theoretical Document Basis at URL http://modis-fire.umd.edu/files/atbd_mod14.pdf. We accessed the MODIS Thermal Anomalies/Fire 5-Min L2 Swath 1-km data (Collection 006) retrieved from Aqua (MYD14) platform from the NASA Fire Information for Resource Management System (FIRMS) (https://earthdata.nasa.gov/earth-observation-data/near-real-time/firms). We use fire detection data flagged with a confidence value in the range 30%-80% and 80%-100% that corresponds to the ‘nominal’ and ‘high’ classes, respectively, as described in the MODIS Collection 6 Fire user guide.

#### MODIS-MAIAC aerosol optical depth

The Multi-Angle Implementation of Atmospheric Correction (MAIAC) algorithm uses time series analysis to retrieve aerosol optical depth and surface bi-directional reflectance factor (BRF) from MODIS measurements over both dark vegetated and bright surfaces^37,38^. For identifying the smoke aerosols generated from biomass burning, MAIAC employs a “smoke test” to discriminate smoke from clouds^39^. The smoke test relies on a relative increase of aerosol absorption at MODIS wavelength 0.412 µm as compared to 0.47–0.67 µm due to multiple scattering and enhanced absorption by organic carbon released during biomass burning combustion. In the present study, we use the best AOD retrievals of MAIAC with some relaxations on the quality flags to capture the extreme smoke loading conditions over the northern India region. MAIAC AOD at 1 km resolution is available as a standard operational MODIS product MCD19A2. A validation analysis of MAIAC using ground-based AERONET AOD measurements at the Kanpur site shown in Supplementary Fig. 2(a) revealed a reasonable agreement with an RMSE and a correlation of 0.23 and 0.86, respectively, suggesting sufficient accuracy of MAIAC aerosol product used to derive long-term trends in AOD over northern India.

#### OMI aerosol absorption optical depth

Near-UV observations from the Ozone Monitoring Instrument (OMI) onboard NASA’s Aura satellite are used to detect UV-absorbing aerosols and measure their optical properties^40^. The UV Aerosol Index (UV-AI) derived from a pair of wavelengths (354–388 nm) qualitatively describes aerosol-led changes in the spectral dependence of the TOA radiances. Positive (negative) values of UV-AI indicate absorbing aerosols (scattering-type aerosols) in the atmosphere; clouds yield near-zero values. The two-channel OMAERUV aerosol algorithm also inverts radiances measured at 354 nm and 388 nm to derive aerosol optical depth and single-scattering albedo simultaneously, thereby aerosol absorption optical depth, at 388 nm on cloud-free pixels. We use the best quality aerosol absorption optical depth retrievals with relaxed criteria in reflectivity (388 nm) to capture heavy smoke loading. The most current version (1.8.9.1) of the OMAERUV product^41^ was obtained from NASA’s GES-DISC data portal at URL https://disc.gsfc.nasa.gov/datasets/OMAERUV_V003/summary.

## Supplementary information

Supplementary Information

## Supplementary information

is available this paper at 10.1038/s41598-019-52799-x.
