# Supplementary material for: Connecting Crop Productivity, Residue Fires, and Air Quality over Northern India
Source: Sci Rep. 2019 Nov 12;9:16594. doi: 10.1038/s41598-019-52799-x (PMC6851147; doi:10.1038/s41598-019-52799-x)
Supplement: Supplementary file 1 — Supplementary Information [file 41598_2019_52799_MOESM1_ESM.pdf]

# Connecting Crop Productivity, Residue Fires, and Air Quality over Northern India

Hiren Jethva[1,2*], Omar Torres[2], Robert D. Field[3],

Alexei Lyapustin[2], Ritesh Gautam[4], Vinay Kayetha[5]

[1]Universities Space Research Association, Columbia, MD 21044 USA

[2]NASA Goddard Space Flight Center, Greenbelt, MD 20771 USA

[3] Columbia University, NASA Goddard Institute for Space Studies, New York, NY 10025 USA

[4]Environmental Defense Fund, Washington, D. C., 20009 USA

[5]Science Systems and Applications, Inc. (SSAI), Lanham, MD 20706 USA

**Mailing Address:**

Room#A422, Building#33,

Laboratory of Atmospheric Chemistry & Dynamics

Earth Science Division

NASA Goddard Space Flight Center,

Greenbelt, MD 20771, USA

**[*]Corresponding Author: Dr. Hiren Jethva**

**E-mail: hiren.t.jethva@nasa.gov**

# SUPPLEMENTAL MATERIAL (BEGINNING FROM NEXT PAGE)

**Supplementary Table 1. Datasets employed in the present study.**

| *Sensors/ Datasets* | *Dataset Characteristics* | *Data Period* |
|---|---|---|
| **Crop area, yield, and production data** | District- and State-wise Crop Production data over India *http://aps.dac.gov.in/APY/Index.htm* | 2002-2016 |
| **MODIS Collection 006** | 1. Aqua-MODIS monthly global Level 3 NDVI Dataset (MYD13C2) https://e4ftl01.cr.usgs.gov/MOLA/ 2. Aqua-MODIS 5-min Level-2 Thermal Anomalies/Fires Product (MYD14) https://earthdata.nasa.gov/earth-observation-data/near-real-time/firms 3. Aqua-MODIS 1-km MAIAC Aerosol Optical Depth (MCD19A2) https://www.nccs.nasa.gov/ | 2002-2016 |
| **OMI** | Level-2 Aerosol product (OMAERUV) Aerosol Absorption Optical Depth https://disc.gsfc.nasa.gov/datasets/OMAERUV_V003/summary | 2004-2016 |
| **MetOne BAM-1020, U.S. Embassy, New Delhi, India** | Hourly measurements of $PM_{2.5}$ https://in.usembassy.gov/embassy-consulates/new-delhi/air-quality-data/ https://www.airnow.gov/ | 2013-2016 |
| **NOAA National Climatic Data Center** | Global Summary of the Day http://www7.ncdc.noaa.gov/CDO/cdo Sub-daily measurements of visibility and related meteorological dataset | 2002-2016 |
| **AERONET Version 3 data** | Spectra Aerosol Optical Depth measurements at Kanpur, India http://aeronet.gsfc.nasa.gov/ | 2002-2016 |

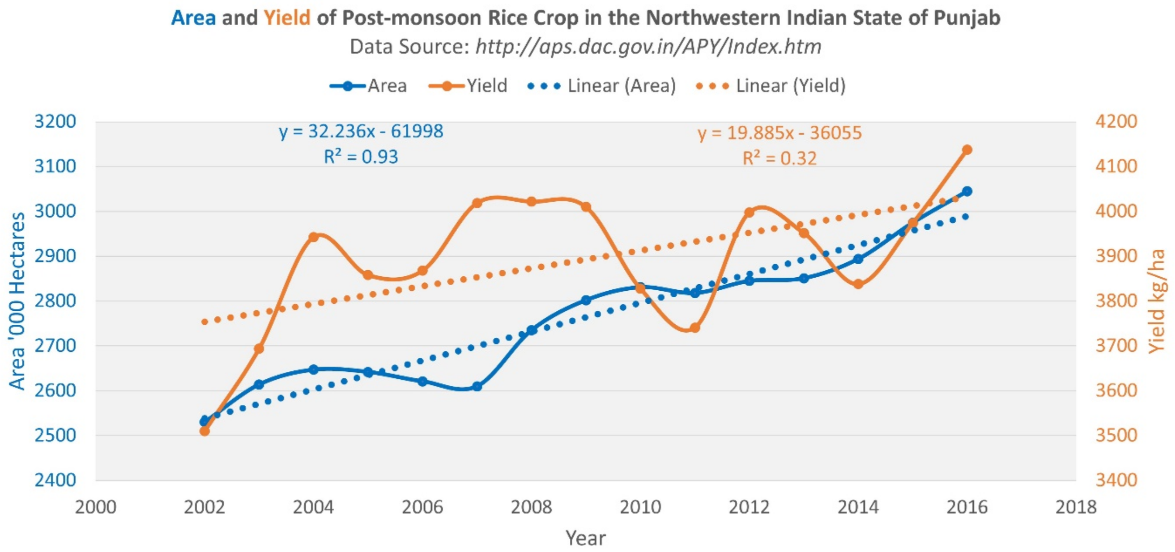


**Supplementary Figure 1** Interannual variations in the area ('000 Hectares) and yield (Kg/hectare) of the post-monsoon rice crop in the northwestern state of Punjab, India. The data were accessed from the Ministry of Agriculture and Farmers Welfare, Govt. of India (http://eands.dacnet.nic.in/).

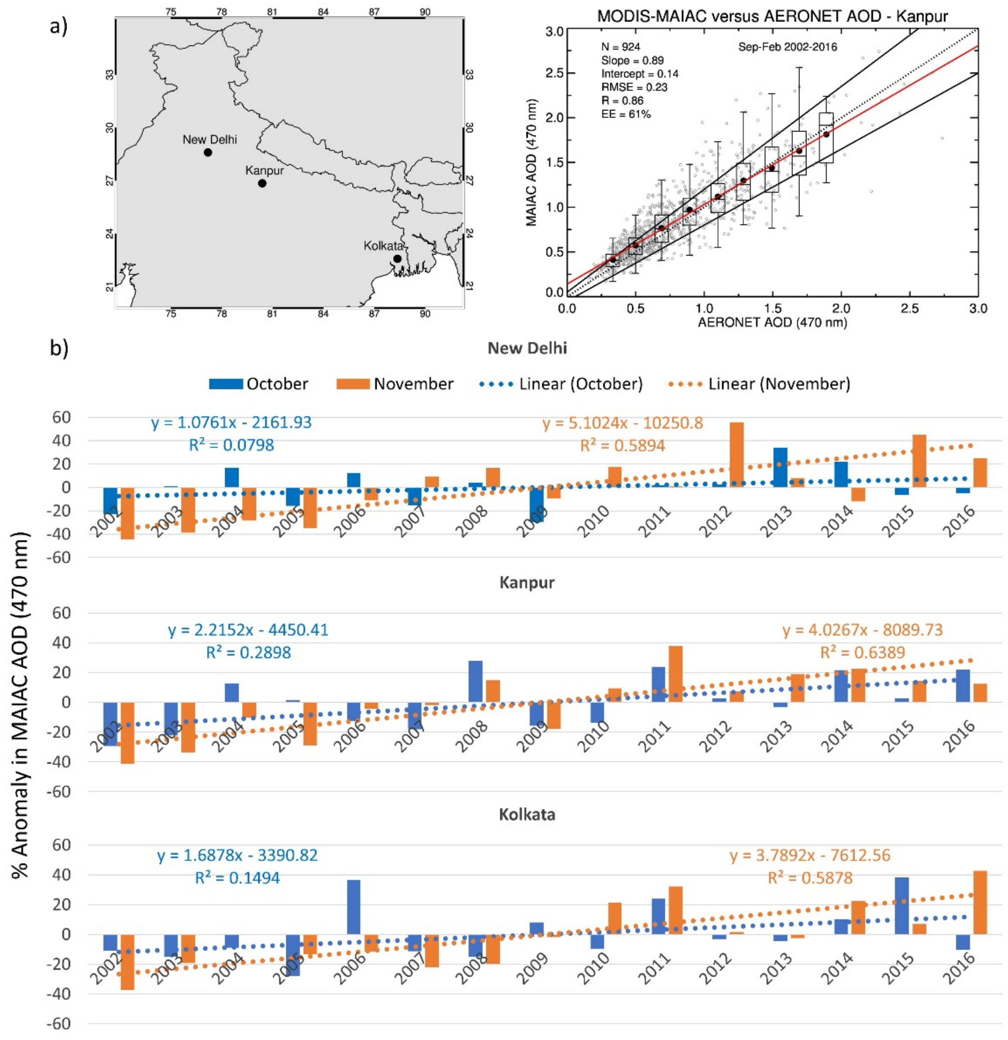


**Supplementary Figure 2 a) A regional map showing locations of three cities in the IGP and validation results of MODIS-MAIAC aerosol optical depth (470 nm) over Kanpur using ground-based AERONET direct measurements**. The comparison was made for measurements made during September through February 2002-2016; resultant statistics are displayed on the top - left. Grey circles are individual matchups, whereas binned data are represented as standard box and whisker format with dark filled circle as mean, horizontal line as median, boxes contains 25 to 75 percentile data, and vertical lines as 1.5 times the interquartile range (25-75 percentile), **b) Interannual changes of the anomaly (%) in MODIS-MAIAC aerosol optical depth (470 nm) for October and November over New Delhi, Kanpur, and Kolkata**. The anomalies (%) were calculated against the long-term averages. Dotted lines represent a linear regression to the multiyear monthly mean data with coefficients of fit are printed within the chart.

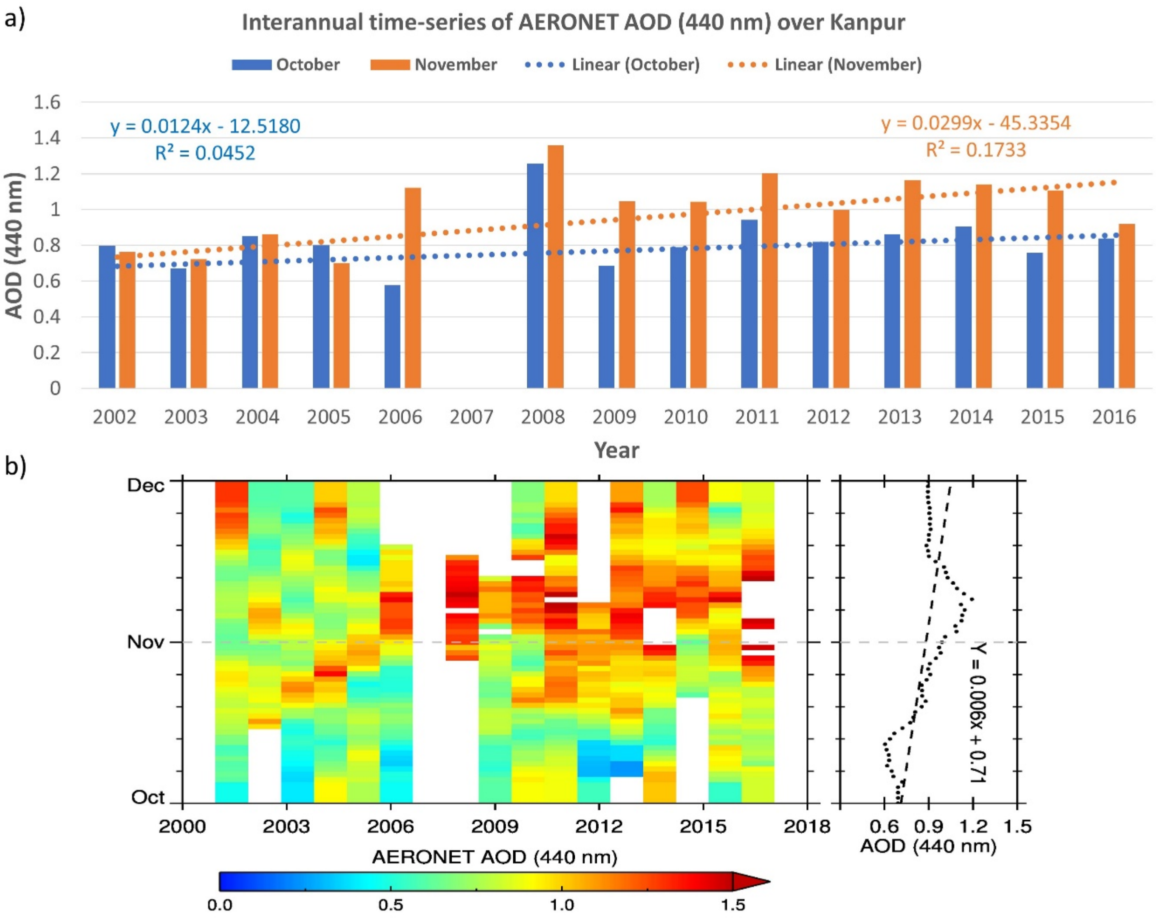


**Supplementary Figure 3 a) Interannual time-series of monthly mean aerosol optical depth (440 nm) for October and November calculated from ground-based AERONET direct measurement data at Kanpur, India**. Dotted lines represent a linear regression to the multiyear monthly mean data with coefficients of fit are printed within the chart. b) A multiyear time-series of 7-day running mean of AERONET AOD (440 nm) for October and November (left) and corresponding averaged AOD and associated trend over Kanpur.

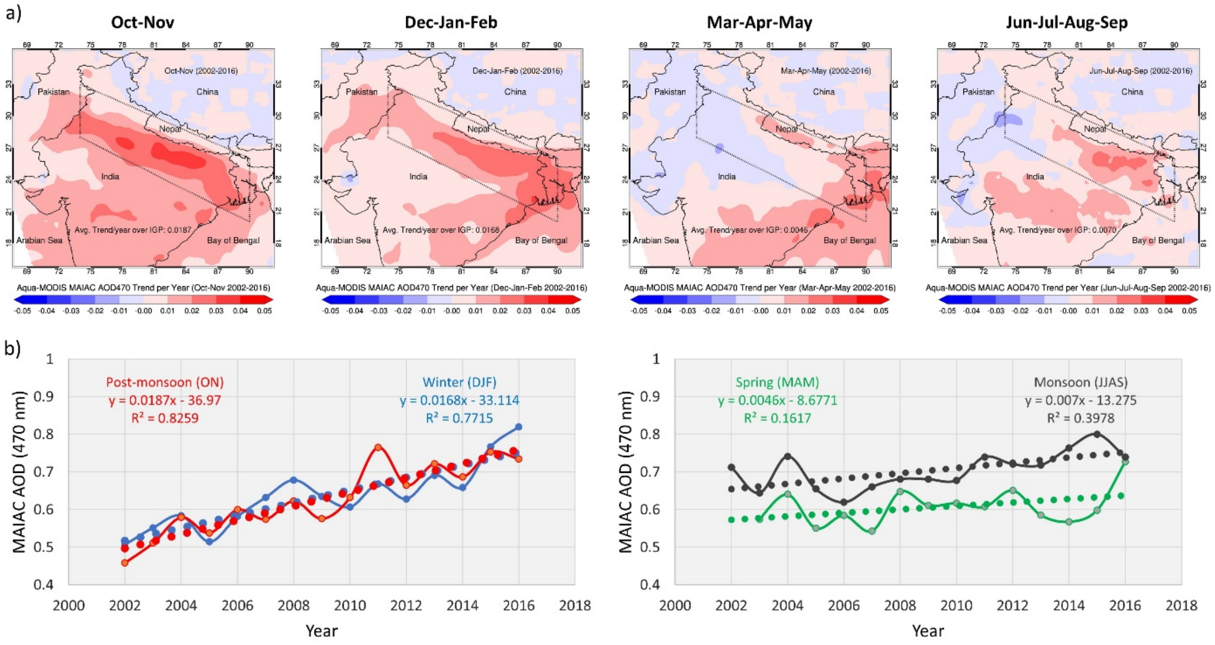


**Supplementary Figure 4 a) Spatial patterns of long-term trends (2002-2016) over most of the Indian subcontinent, and b) multi-year time-series over the IGP region of Aqua/MODIS MAIAC AOD (470 nm) for different seasons**. The IGP area bounded by a dotted box is considered for calculating the area-averaged trends depicted in the respective plots.
